# (2*E*)-1-(2-Hy­droxy-5-methyl­phen­yl)-3-(4-meth­oxy­phen­yl)prop-2-en-1-one

**DOI:** 10.1107/S1600536811015054

**Published:** 2011-04-29

**Authors:** Hoong-Kun Fun, Suhana Arshad, B. K. Sarojini, V. Musthafa Khaleel, B. Narayana

**Affiliations:** a X-ray Crystallography Unit, School of Physics, Universiti Sains Malaysia, 11800 USM, Penang, Malaysia; b Department of Chemistry, P. A. College of Engineering, Mangalore 574 153, India; c Department of Studies in Chemistry, Mangalore University, Mangalagangotri, Mangalore 574 199, India.

## Abstract

In the title compound, C_17_H_16_O_3_, the dihedral angle between the aromatic rings is 4.59 (7)° and an intra­molecular O—H⋯O hydrogen bond generates an *S*(6) ring. In the crystal, adjacent mol­ecules are linked by C—H⋯O hydrogen bonds, leading to the formation of [001] supra­molecular chains. Weak C—H⋯π inter­actions consolidate the packing.

## Related literature

For a related structure and background references to chalcones, see: Fun *et al.* (2010 ▶). For related structures, see: Chantrapromma *et al.* (2009 ▶, 2010 ▶); Fun *et al.* (2009 ▶); Horkaew *et al.* (2010 ▶); Lu *et al.* (2009 ▶); Suwunwong *et al.* (2009 ▶); Wang *et al.* (2009 ▶, 2010 ▶); Jasinski *et al.* (2011 ▶). For hydrogen-bond motifs, see: Bernstein *et al.* (1995 ▶). For bond-length data, see: Allen *et al.* (1987 ▶).
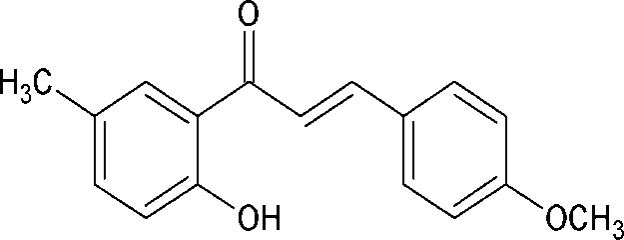


## Experimental

### 

#### Crystal data

C_17_H_16_O_3_
*M* *_r_* = 268.30
Monoclinic, 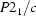

*a* = 12.6990 (18) Å
*b* = 8.8022 (13) Å
*c* = 13.172 (2) Å
β = 105.565 (2)°
*V* = 1418.3 (4) Å^3^
*Z* = 4
Mo *K*α radiation
μ = 0.09 mm^−1^
*T* = 296 K
0.46 × 0.32 × 0.18 mm

#### Data collection

Bruker SMART APEXII DUO CCD diffractometer
Absorption correction: multi-scan (*SADABS*; Bruker, 2009 ▶) *T*_min_ = 0.962, *T*_max_ = 0.984
11493 measured reflections
4090 independent reflections
2608 reflections with *I* > 2σ(*I*)
*R*_int_ = 0.024

#### Refinement

*R*[*F* ^2^ > 2σ(*F* ^2^)] = 0.047
*wR*(*F* ^2^) = 0.131
*S* = 1.02
4090 reflections
187 parameters
H atoms treated by a mixture of independent and constrained refinement
Δρ_max_ = 0.16 e Å^−3^
Δρ_min_ = −0.16 e Å^−3^

### 

Data collection: *APEX2* (Bruker, 2009 ▶); cell refinement: *SAINT* (Bruker, 2009 ▶); data reduction: *SAINT*; program(s) used to solve structure: *SHELXTL* (Sheldrick, 2008 ▶); program(s) used to refine structure: *SHELXTL*; molecular graphics: *SHELXTL*; software used to prepare material for publication: *SHELXTL* and *PLATON* (Spek, 2009 ▶).

## Supplementary Material

Crystal structure: contains datablocks global, I. DOI: 10.1107/S1600536811015054/hb5847sup1.cif

Structure factors: contains datablocks I. DOI: 10.1107/S1600536811015054/hb5847Isup2.hkl

Supplementary material file. DOI: 10.1107/S1600536811015054/hb5847Isup3.cml

Additional supplementary materials:  crystallographic information; 3D view; checkCIF report

## Figures and Tables

**Table 1:** Hydrogen-bond geometry (Å, °) *Cg*1 is the centroid of the C1–C6 ring.

*D*—H⋯*A*	*D*—H	H⋯*A*	*D*⋯*A*	*D*—H⋯*A*
O1—H1*O*1⋯O2	0.95 (2)	1.65 (2)	2.5112 (18)	149.4 (19)
C11—H11*A*⋯O3^i^	0.93	2.60	3.4317 (17)	149
C16—H16*C*⋯*Cg*1^ii^	0.96	2.81	3.5800 (18)	138

Symmetry codes: (i) 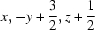; (ii) 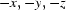.

**Fig. 1.**
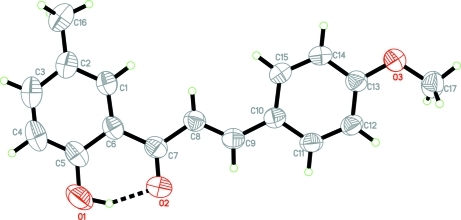
The molecular structure of the title compound, showing 50% probability displacement ellipsoids. The dashed line indicates the intramolecular bond.

**Fig. 2.**
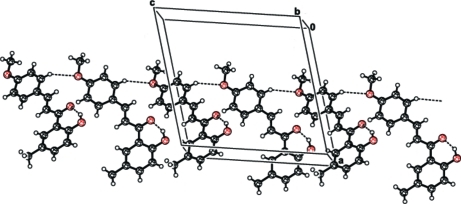
The crystal packing of the title compound, viewed along the b axis.

## supplementary crystallographic information

### Comment

In continuation of our studies on the crystal structures of chalcones (Fun *et al.*, 2010), we now report the synthesis and crystal structure of the title compound, (I). The structures of some related chalcones *viz*: (*Z*)-3-(9-anthryl)-1-(4-methoxyphenyl)prop-2-en-1-one (Chantrapromma *et al.*, 2009), (*Z*)-3-(9-anthryl)- 1-(2-thienyl)prop-2-en-1-one (Fun *et al.*, 2009), (*E*)-3- (anthracen-9-yl)-1-(4-bromophenyl)prop-2-en-1-one (Suwunwong *et al.*, 2009), (*Z*)-3-(9-anthryl)-1-(4-bromophenyl)-2-(4-nitro-1*H*- imidazol-1-yl)prop-2-en-1-one (Lu *et al.*, 2009),(*Z*)-3- (9-anthryl)-2-(4-nitro-1*H*-imidazol-1-yl)-1-*p*-tolylprop- 2-en-1-one (Wang *et al.*, 2009), (*E*)-3-(9-anthryl)-1- (4-fluorophenyl)-2-(4-nitro-1*H*-imidazol-1-yl)prop-2-en-1-one (Wang *et al.*, 2010), (*E*)-3-(anthracen-9-yl)-1-(furan-2-yl) prop-2-en-1-one (Horkaew *et al.*, 2010), and an orthorhombic polymorph of (*Z*)-3-(9-anthryl)-1-(2-thienyl)prop-2-en-1-one (Chantrapromma *et al.*, 2010) and 2(*E*)-3-(4-hydroxyphenyl)-1-(4-chlorophenyl) prop-2-en-1-one (Jasinski *et al.*,2011) have been reported.

The molecular structure is shown in Fig. 1. An intramolecular O1—H1O1···O2 hydrogen bond (Table 1) stabilizes the molecular structure and forms an *S*(6) ring motif (Bernstein *et al.*, 1995). The dihedral angle between the phenyl (C1–C6) ring and the methoxy-substituted phenyl (C10–C15) ring is 4.59 (7)°. Bond lengths (Allen *et al.*, 1987) and angles are within normal ranges and are comparable to the related structures (Fun *et al.*, 2010).

In the crystal packing (Fig. 2), the molecules are linked into infinite one-dimensional chain along the *c*-axis by intermolecular C11—H11A···O3 hydrogen bonds (Table 1). There are also C—H···π interactions (Table 1) which involves C16 and phenyl ring (*Cg*1 = C1–C6).

### Experimental

2-Hydroxy-5-methylacetophenone (1.50 g, 0.01 mol) was mixed with 4-methoxybenzaldehyde (1.36 g, 0.01 mol) and dissolved in ethanol (40 ml). To this solution, 5 ml of KOH (50%) was added at 278 K. The reaction mixture stirred for 6 h and poured on to crushed ice. The pH of this mixture was adjusted to 3–4 with 2 *M* HCl aqueous solution. The resulting crude yellow solid was filtered, washed successively with dilute HCl solution and distilled water and finally recrystallized from ethanol (95%) to give the pure chalcone.  Orange blocks of (I) were grown by the slow evaporation of the solution of the compound in ethyl alcohol (*m. p.*: 361 K). Composition: Found (Calculated) for C_17_H_16_O_3_, C: 76.10 (76.16); H: 6.01 (6.05).

### Refinement

H1O1 atom attached to the O atom was located from the difference map and refined freely [O–H = 0.94 (2) Å]. The remaining H atoms were positioned geometrically [C–H = 0.93 or 0.96 Å] and refined using a riding model with *U*_iso_(H) = 1.2 or 1.5 *U*_eq_(C). A rotating group model was applied to the methyl groups.

### Crystal data

**Table:**

C_17_H_16_O_3_	*F*(000) = 568
*M**_r_* = 268.30	*D*_x_ = 1.256 Mg m^−^^3^
Monoclinic, *P*2_1_/*c*	Mo *K*α radiation, λ = 0.71073 Å
Hall symbol: -P 2ybc	Cell parameters from 2769 reflections
*a* = 12.6990 (18) Å	θ = 2.8–28.6°
*b* = 8.8022 (13) Å	µ = 0.09 mm^−^^1^
*c* = 13.172 (2) Å	*T* = 296 K
β = 105.565 (2)°	Block, orange
*V* = 1418.3 (4) Å^3^	0.46 × 0.32 × 0.18 mm
*Z* = 4	

### Data collection

**Table:**

Bruker SMART APEXII DUO CCD diffractometer	4090 independent reflections
Radiation source: fine-focus sealed tube	2608 reflections with *I* > 2σ(*I*)
graphite	*R*_int_ = 0.024
φ and ω scans	θ_max_ = 29.9°, θ_min_ = 2.8°
Absorption correction: multi-scan (*SADABS*; Bruker, 2009)	*h* = −17→17
*T*_min_ = 0.962, *T*_max_ = 0.984	*k* = −10→12
11493 measured reflections	*l* = −18→18

### Refinement

**Table:**

Refinement on *F*^2^	Primary atom site location: structure-invariant direct methods
Least-squares matrix: full	Secondary atom site location: difference Fourier map
*R*[*F*^2^ > 2σ(*F*^2^)] = 0.047	Hydrogen site location: inferred from neighbouring sites
*wR*(*F*^2^) = 0.131	H atoms treated by a mixture of independent and constrained refinement
*S* = 1.02	*w* = 1/[σ^2^(*F*_o_^2^) + (0.0547*P*)^2^ + 0.1652*P*] where *P* = (*F*_o_^2^ + 2*F*_c_^2^)/3
4090 reflections	(Δ/σ)_max_ < 0.001
187 parameters	Δρ_max_ = 0.16 e Å^−^^3^
0 restraints	Δρ_min_ = −0.16 e Å^−^^3^

### Special details

**Table:**

Geometry. All e.s.d.'s (except the e.s.d. in the dihedral angle between two l.s. planes) are estimated using the full covariance matrix. The cell e.s.d.'s are taken into account individually in the estimation of e.s.d.'s in distances, angles and torsion angles; correlations between e.s.d.'s in cell parameters are only used when they are defined by crystal symmetry. An approximate (isotropic) treatment of cell e.s.d.'s is used for estimating e.s.d.'s involving l.s. planes.
Refinement. Refinement of *F*^2^ against ALL reflections. The weighted *R*-factor *wR* and goodness of fit *S* are based on *F*^2^, conventional *R*-factors *R* are based on *F*, with *F* set to zero for negative *F*^2^. The threshold expression of *F*^2^ > σ(*F*^2^) is used only for calculating *R*-factors(gt) *etc*. and is not relevant to the choice of reflections for refinement. *R*-factors based on *F*^2^ are statistically about twice as large as those based on *F*, and *R*- factors based on ALL data will be even larger.

### Fractional atomic coordinates and isotropic or equivalent isotropic displacement parameters (Å^2^)

**Table:**

	*x*	*y*	*z*	*U*_iso_*/*U*_eq_	
O1	0.13821 (11)	0.18686 (15)	0.32930 (8)	0.0794 (4)	
O2	0.26594 (9)	0.35672 (14)	0.26545 (7)	0.0758 (3)	
O3	0.51456 (8)	0.87081 (13)	−0.15126 (7)	0.0666 (3)	
C1	0.03317 (10)	0.26022 (14)	0.04622 (10)	0.0486 (3)	
H1A	0.0518	0.3125	−0.0078	0.058*	
C2	−0.06268 (11)	0.17724 (16)	0.02260 (12)	0.0553 (3)	
C3	−0.08898 (13)	0.10217 (19)	0.10552 (15)	0.0704 (4)	
H3A	−0.1538	0.0472	0.0921	0.084*	
C4	−0.02277 (15)	0.10664 (19)	0.20568 (15)	0.0728 (4)	
H4A	−0.0427	0.0543	0.2590	0.087*	
C5	0.07432 (12)	0.18853 (16)	0.22906 (11)	0.0581 (4)	
C6	0.10376 (10)	0.26897 (14)	0.14840 (10)	0.0468 (3)	
C7	0.20644 (11)	0.35632 (16)	0.17338 (10)	0.0502 (3)	
C8	0.23943 (10)	0.44232 (15)	0.09183 (10)	0.0488 (3)	
H8A	0.1929	0.4469	0.0240	0.059*	
C9	0.33518 (10)	0.51410 (15)	0.11356 (10)	0.0478 (3)	
H9A	0.3790	0.5037	0.1822	0.057*	
C10	0.38013 (9)	0.60674 (14)	0.04375 (9)	0.0442 (3)	
C11	0.48166 (10)	0.67433 (16)	0.08326 (10)	0.0501 (3)	
H11A	0.5188	0.6589	0.1536	0.060*	
C12	0.52938 (10)	0.76377 (16)	0.02169 (10)	0.0504 (3)	
H12A	0.5973	0.8081	0.0504	0.060*	
C13	0.47519 (10)	0.78657 (15)	−0.08275 (9)	0.0477 (3)	
C14	0.37276 (11)	0.72165 (17)	−0.12373 (10)	0.0581 (4)	
H14A	0.3355	0.7385	−0.1939	0.070*	
C15	0.32612 (10)	0.63348 (16)	−0.06231 (10)	0.0542 (3)	
H15A	0.2577	0.5907	−0.0912	0.065*	
C16	−0.13460 (12)	0.16543 (19)	−0.08791 (14)	0.0704 (4)	
H16A	−0.1021	0.2204	−0.1347	0.106*	
H16B	−0.2051	0.2077	−0.0911	0.106*	
H16C	−0.1427	0.0606	−0.1086	0.106*	
C17	0.61893 (12)	0.93974 (19)	−0.11419 (12)	0.0645 (4)	
H17A	0.6362	0.9942	−0.1708	0.097*	
H17B	0.6731	0.8627	−0.0886	0.097*	
H17C	0.6181	1.0090	−0.0581	0.097*	
H1O1	0.2008 (17)	0.245 (2)	0.3290 (16)	0.107 (7)*	

### Atomic displacement parameters (Å^2^)

**Table:**

	*U*^11^	*U*^22^	*U*^33^	*U*^12^	*U*^13^	*U*^23^
O1	0.1019 (9)	0.0879 (9)	0.0533 (6)	−0.0055 (7)	0.0295 (6)	0.0145 (6)
O2	0.0760 (7)	0.1013 (9)	0.0451 (5)	−0.0174 (6)	0.0078 (5)	0.0079 (5)
O3	0.0642 (6)	0.0840 (8)	0.0509 (5)	−0.0187 (5)	0.0142 (5)	0.0088 (5)
C1	0.0523 (7)	0.0438 (7)	0.0542 (7)	0.0022 (6)	0.0222 (6)	−0.0008 (6)
C2	0.0514 (7)	0.0456 (8)	0.0729 (9)	0.0012 (6)	0.0239 (6)	−0.0062 (6)
C3	0.0651 (9)	0.0595 (10)	0.0961 (13)	−0.0098 (7)	0.0380 (9)	−0.0011 (9)
C4	0.0863 (11)	0.0626 (10)	0.0845 (11)	−0.0079 (9)	0.0488 (10)	0.0088 (8)
C5	0.0756 (9)	0.0509 (8)	0.0567 (8)	0.0042 (7)	0.0331 (7)	0.0038 (6)
C6	0.0552 (7)	0.0413 (7)	0.0496 (7)	0.0036 (6)	0.0241 (6)	−0.0015 (5)
C7	0.0570 (7)	0.0503 (8)	0.0454 (6)	0.0025 (6)	0.0172 (6)	−0.0010 (5)
C8	0.0537 (7)	0.0499 (7)	0.0428 (6)	−0.0015 (6)	0.0130 (5)	−0.0013 (5)
C9	0.0503 (6)	0.0489 (7)	0.0435 (6)	0.0024 (6)	0.0114 (5)	−0.0028 (5)
C10	0.0445 (6)	0.0453 (7)	0.0427 (6)	0.0028 (5)	0.0113 (5)	−0.0040 (5)
C11	0.0458 (6)	0.0617 (9)	0.0395 (6)	0.0009 (6)	0.0058 (5)	0.0004 (6)
C12	0.0400 (6)	0.0621 (9)	0.0462 (6)	−0.0029 (6)	0.0065 (5)	−0.0027 (6)
C13	0.0483 (6)	0.0521 (8)	0.0434 (6)	−0.0004 (6)	0.0135 (5)	−0.0012 (5)
C14	0.0554 (7)	0.0723 (10)	0.0401 (6)	−0.0100 (7)	0.0016 (6)	0.0034 (6)
C15	0.0465 (7)	0.0632 (9)	0.0481 (7)	−0.0098 (6)	0.0042 (5)	−0.0018 (6)
C16	0.0544 (8)	0.0657 (10)	0.0876 (11)	−0.0045 (7)	0.0131 (8)	−0.0117 (8)
C17	0.0614 (8)	0.0662 (10)	0.0707 (9)	−0.0104 (7)	0.0257 (7)	−0.0025 (7)

### Geometric parameters (Å, °)

**Table:**

O1—C5	1.3517 (18)	C9—C10	1.4554 (17)
O1—H1O1	0.94 (2)	C9—H9A	0.9300
O2—C7	1.2449 (15)	C10—C11	1.3881 (17)
O3—C13	1.3624 (15)	C10—C15	1.4011 (17)
O3—C17	1.4198 (17)	C11—C12	1.3814 (18)
C1—C2	1.3815 (18)	C11—H11A	0.9300
C1—C6	1.4048 (18)	C12—C13	1.3778 (17)
C1—H1A	0.9300	C12—H12A	0.9300
C2—C3	1.392 (2)	C13—C14	1.3903 (18)
C2—C16	1.500 (2)	C14—C15	1.3656 (19)
C3—C4	1.361 (2)	C14—H14A	0.9300
C3—H3A	0.9300	C15—H15A	0.9300
C4—C5	1.390 (2)	C16—H16A	0.9600
C4—H4A	0.9300	C16—H16B	0.9600
C5—C6	1.4082 (18)	C16—H16C	0.9600
C6—C7	1.4729 (18)	C17—H17A	0.9600
C7—C8	1.4641 (18)	C17—H17B	0.9600
C8—C9	1.3315 (17)	C17—H17C	0.9600
C8—H8A	0.9300		
			
C5—O1—H1O1	106.0 (13)	C11—C10—C9	119.05 (11)
C13—O3—C17	118.65 (11)	C15—C10—C9	123.64 (11)
C2—C1—C6	122.82 (12)	C12—C11—C10	122.26 (11)
C2—C1—H1A	118.6	C12—C11—H11A	118.9
C6—C1—H1A	118.6	C10—C11—H11A	118.9
C1—C2—C3	117.18 (14)	C13—C12—C11	119.26 (11)
C1—C2—C16	121.70 (13)	C13—C12—H12A	120.4
C3—C2—C16	121.11 (14)	C11—C12—H12A	120.4
C4—C3—C2	122.06 (15)	O3—C13—C12	124.53 (11)
C4—C3—H3A	119.0	O3—C13—C14	115.97 (11)
C2—C3—H3A	119.0	C12—C13—C14	119.50 (12)
C3—C4—C5	120.69 (14)	C15—C14—C13	120.86 (12)
C3—C4—H4A	119.7	C15—C14—H14A	119.6
C5—C4—H4A	119.7	C13—C14—H14A	119.6
O1—C5—C4	118.37 (13)	C14—C15—C10	120.80 (12)
O1—C5—C6	122.08 (14)	C14—C15—H15A	119.6
C4—C5—C6	119.54 (14)	C10—C15—H15A	119.6
C1—C6—C5	117.70 (12)	C2—C16—H16A	109.5
C1—C6—C7	122.80 (11)	C2—C16—H16B	109.5
C5—C6—C7	119.49 (12)	H16A—C16—H16B	109.5
O2—C7—C8	119.66 (12)	C2—C16—H16C	109.5
O2—C7—C6	119.25 (12)	H16A—C16—H16C	109.5
C8—C7—C6	121.09 (11)	H16B—C16—H16C	109.5
C9—C8—C7	120.81 (12)	O3—C17—H17A	109.5
C9—C8—H8A	119.6	O3—C17—H17B	109.5
C7—C8—H8A	119.6	H17A—C17—H17B	109.5
C8—C9—C10	128.35 (12)	O3—C17—H17C	109.5
C8—C9—H9A	115.8	H17A—C17—H17C	109.5
C10—C9—H9A	115.8	H17B—C17—H17C	109.5
C11—C10—C15	117.30 (12)		
			
C6—C1—C2—C3	1.0 (2)	O2—C7—C8—C9	3.8 (2)
C6—C1—C2—C16	−177.83 (13)	C6—C7—C8—C9	−176.22 (12)
C1—C2—C3—C4	−1.3 (2)	C7—C8—C9—C10	−178.59 (12)
C16—C2—C3—C4	177.45 (14)	C8—C9—C10—C11	178.78 (13)
C2—C3—C4—C5	0.5 (3)	C8—C9—C10—C15	−0.7 (2)
C3—C4—C5—O1	−178.70 (15)	C15—C10—C11—C12	−0.5 (2)
C3—C4—C5—C6	0.7 (2)	C9—C10—C11—C12	−179.97 (12)
C2—C1—C6—C5	0.22 (19)	C10—C11—C12—C13	−0.4 (2)
C2—C1—C6—C7	179.43 (12)	C17—O3—C13—C12	0.6 (2)
O1—C5—C6—C1	178.32 (12)	C17—O3—C13—C14	−179.83 (13)
C4—C5—C6—C1	−1.1 (2)	C11—C12—C13—O3	−179.21 (13)
O1—C5—C6—C7	−0.9 (2)	C11—C12—C13—C14	1.2 (2)
C4—C5—C6—C7	179.72 (13)	O3—C13—C14—C15	179.21 (13)
C1—C6—C7—O2	−178.73 (13)	C12—C13—C14—C15	−1.2 (2)
C5—C6—C7—O2	0.47 (19)	C13—C14—C15—C10	0.3 (2)
C1—C6—C7—C8	1.27 (19)	C11—C10—C15—C14	0.5 (2)
C5—C6—C7—C8	−179.54 (12)	C9—C10—C15—C14	179.98 (13)

### Hydrogen-bond geometry (Å, °)

**Table:**

*D*—H···*A*	*D*—H	H···*A*	*D*···*A*	*D*—H···*A*
O1—H1O1···O2	0.95 (2)	1.65 (2)	2.5112 (18)	149.4 (19)
C11—H11A···O3^i^	0.93	2.60	3.4317 (17)	149.
C16—H16C···Cg1^ii^	0.96	2.81	3.5800 (18)	138

Symmetry codes: (i) *x*, −*y*+3/2, *z*+1/2; (ii) −*x*, −*y*, −*z*.
